# Is Teething an Illness?

**Published:** 1913-01-11

**Authors:** 


### MATERNITY AND MOTHERING.

**(Inquiries and Correspondence are invited.)**

### Is Teething an Illness?

If there is one conviction held more profoundly by mothers and nurses in regard to babies, it is that numberless disorders of infant life are due to the process of "cutting the teeth." Any illness, whether of the most trivial or the most serious nature, occurring in any child between four months and three years is invariably ascribed to the mystical but soul-satisfying fetish of "teething." Be it tuberculous meningitis, be it whooping-cough, fits, colic, bad temper, or what not, any and every disorder within those age limits of babydom can be explained by that blessed word.

The present writer has come across three instances within a few days in which comparatively well educated and enlightened mothers and nurses have ascribed to teething a cold, an attack of diarrhœa, and a skin eruption respectively. The latter was in fact a "napkin-rash," solely due to irritation of the skin by wetted pilches which should have been more frequently changed. It disappeared in forty-eight hours under liberal inunction with a simple ointment and more frequent changes; but the nurse-in-charge is still quite certain that it was caused by the teeth, which in this particular case had not begun to appear. So, too, "red-gum" is an article of faith with most mothers. This is the name given to any rash, whatever its character, which arises in an infant; needless to say, the cause of red-gum is the infallible "teething."

The medical profession in olden days was doubtless responsible for setting up this extraordinary idol for maternal worship. In 1732 an English author said that 10 per cent. of all children died of "teething." In 1842 the same all embracing complaint was the certified cause of death in 5 per cent. of the infant mortality in London. In 1886 there were 4,900 deaths registered as due to teething in England and Wales; and even at the present day there are still a few practitioners who are content to enter the word fairly frequently on their baby patients' death certificates. It is, therefore, not surprising that the existence of a serious disease of this name should be accepted as an article of faith by the vast majority of the laity.

Nevertheless, we make bold to say that this supposed disease has no existence in fact at all. Further, we assert that the conception of teething as a *direct* cause of ill-health has been the cause of infantile sickness and death, the magnitude of which it would be difficult to over-estimate. A recent writer * on this topic classifies four distinct shades of opinion among professional men on the subject. First, the rapidly diminishing band of those who regard dentition, like the average mother and nurse, as a fertile source of all kinds of symptoms and illnesses. These are now all but extinct within the profession. Next, those who have a vague notion that the process of cutting teeth does somehow predispose to other diseases setting up irritability of the nervous system or a lessened resisting power, or reflex disturbances of some kind. Thirdly, those who have emancipated themselves from the shibboleths of the first class, but find it convenient to retain the term as a means of pacifying anxious mothers and of satisfying the insatiable cravings of their minds for some explanation of their babies' ailments which shall reflect in no wise upon anyone except Providence.

### An Australian View.

The fourth class, in Dr. Hone's own words, "regard dentition as having absolutely nothing to do with any illness, beyond possibly one or two local conditions of the mouth, and with these only indirectly. They further believe that on account of the untold harm that has resulted in the past to infants from the fetish of teething, from the undoubted influence the delusion has in increasing infantile mortality, and from the support it gives to all sorts of quackery in the way of teething collars, teething powders, and similar abominations, the term ought to be swept out of the medical dictionary as having any relation to morbid conditions."

This is a sweeping denunciation, but any experienced practitioner who thinks for himself could quote a formidable array of facts in support of it. Children, especially babies, are apt to suffer from all sorts of illnesses and ailments whose exact origin is obscure. The half-hearted clinician, who

* Dr. F. S. Hone, *Australasian Medical Gazette*, October 19, 1912, p. 400.

accepts the mother's glib explanation of teething without taking the trouble to investigate matters thoroughly for himself, will fail to do his duty to his patients and will make many errors in diagnosis. The more thoroughly a case of illness in a child is gone into the greater is the chance of an accurate diagnosis being made. Fretfulness, rise of temperature, lassitude, and *malaise* in a baby should be the signal for a most searching investigation until the trouble has been tracked to its origin. He who will do this properly will seldom have to fall back upon mythical diagnosis such as teething for an explanation of the case.

Nor is this iconoclastic view lacking in official confirmation. At the British Medical Association meeting of 1908 the matter was discussed thoroughly, and the consensus was very much in accord with the position here outlined. At the same time it has to be recognised that there are some first-class authorities who do not subscribe to it without reservation. It suffices to mention Dr. G. F. Still, who still defends the view that numerous diseases, including bronchitis and diarrhœa, as well as nervous conditions, may arise partly or wholly through "teething." Holt, the leading authority in the United States, also is disposed to admit that in exceptional cases the cutting of teeth is signalised by symptoms of ill-health; though he does not go nearly so far as Still in this respect. There is one fairly satisfactory way of reconciling these differences of opinion. The second six months of life are especially a time of dietary changes and experiments. The nursling at this stage begins to forsake a diet of milk only, and to consume other less digestible fare. Little wonder that in some cases temporary digestive disturbances arise, including colic, diarrhœa, vomiting, and so forth. And similarly may other more remote consequences follow secondarily nutritional processes thus disturbed, such as coughs, colds, fits, bronchitis, etc.

The Practical Lesson.

To argue the whole matter out is foreign to our present purpose, and would involve too much technicality. Suffice it to emphasise once more the view that teething is no more a *direct* cause of rashes, digestive disturbances, cough, or any other illness than of a broken leg; it may coincide with any of those troubles, because a baby between six months and two years is pretty certain to have recently cut a tooth or to be about to cut another. Neglect to procure expert advice, on the ground that the trouble is an inevitable result of teething, is only too common, and has resulted in calamities and disasters innumerable which might have been prevented. This saying will seem a hard one to many who have had the care of the children, and cherish the superstition in question without ever having critically examined it. But we believe it to be true, or at least to have so few exceptions that as a general rule of conduct it is the only safe one to adopt. If only mothers and nurses would believe it and follow it, the next generation would certainly have a better chance of health and strength. Perhaps by the time this century is middle aged they will; and the laity, as well as the doctors, will regard the views of their ancestors with the same amused contempt that we now regard the fleams and leeches of the Middle Ages. One thing at least we can already be thankful for; and that is that the practice of "lancing the gums"—itself the natural outcome of the old-fashioned views about teething—is already as dead as Queen Anne. This fact alone suffices to show that the profession has found salvation, and in time the same gospel will filter through to the laity.

